# Lung-targeting lentiviral vector for passive immunisation against influenza

**DOI:** 10.1136/thoraxjnl-2020-214656

**Published:** 2020-09-03

**Authors:** Tiong Kit Tan, Toby P E Gamlen, Pramila Rijal, Alain R Townsend, Deborah R Gill, Stephen C Hyde

**Affiliations:** 1 Radcliffe Department of Medicine, Gene Medicine Research Group, Nuffield Division of Clinical Laboratory Science, Oxford, UK; 2 Radcliffe Department of Medicine, MRC Human Immunology Unit, MRC Weatherall Institute of Molecular Medicine, Oxford, UK; 3 Gene Medicine Research Group, Nuffield Division of Clinical Laboratory Sciences, Radcliffe Department of Medicine, Oxford University, Oxford, UK; 4 UK Cystic Fibrosis Gene Therapy Consortium, Oxford, UK

**Keywords:** viral infection, respiratory infection, infection control

## Abstract

When recombinant simian immunodeficiency virus (SIV) is pseudotyped with the F and HN glycoproteins from murine respiratory Sendai virus (rSIV.F/HN), it provides efficient lung cell targeting and lifelong transgene expression in the murine airways. We have shown that a single dose of rSIV.F/HN can direct stable expression of neutralising antibody against influenza in the murine airways and systemic circulation, and protects mice against two different influenza strains in lethal challenge experiments. These data suggest that rSIV.F/HN could be used as a vector for passive immunisation against influenza and other respiratory pathogens.

## Introduction

Seasonal influenza is a major worldwide health risk resulting in the deaths of 300 000–650 000 people annually,^1^ with the potential for pandemic influenza to lead to an even greater number. Neither prior infections, nor current inactivated vaccines, provide effective and lasting protection against influenza infection due to the rapid antigenic evolution of the virus. One of the many strategies being evaluated to protect against influenza is the use of recombinant viral vectors to deliver transgenes expressing neutralising anti-influenza antibody (nAb) to target tissues. Recombinant Adeno-associated virus (rAAV) vectors have been shown to direct expression of antibody that confers protection against influenza in animal models following intramuscular^2^ and intranasal delivery.^3^ Here, we report the use of a lung-targeting lentiviral vector to direct the expression of nAb T1-3B^4^ in the murine airways. The recombinant simian immunodeficiency virus (SIV) is pseudotyped with the F and HN surface glycoproteins from Sendai virus^5^ generating efficient airway tropism, and abundant, lifelong transgene expression in the murine airways without evidence of toxicity.^6–8^ This rSIV.F/HN vector is being evaluated for treatment of cystic fibrosis lung disease and also offers the potential for repeated administration^6^ which is a major problem of rAAV.^9^ We hypothesised that rSIV.F/HN could direct expression of nAb in the murine airways to provide passive immunity to influenza infection and protect mice against supralethal influenza challenge.

## Methods

Detailed methods can be found in the online supplementary information.

10.1136/thoraxjnl-2020-214656.supp1 Supplementary data

## Results

### rSIV.F/HN mediates expression of anti-influenza nAb in the murine airways

**Figure 1:**
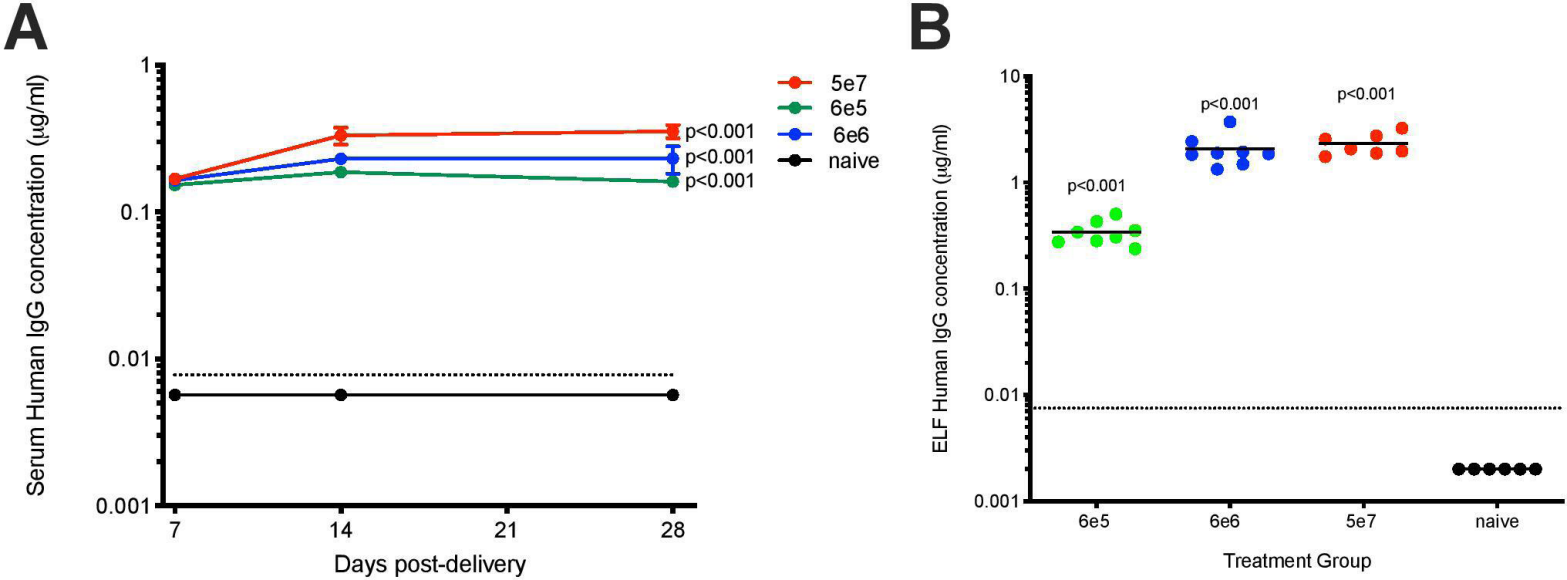
Intranasal delivery of rSIV.F/HN vector can lead to robust antibody expression in the murine lung lumen and serum. Mice (BALB/c, n=6–8 per group) were dosed with rSIV.F/HN.hCEF.T13B (6e5, 6e6 or 5e7 TU/mouse) via nasal instillation or remained naive. Serum was collected at the indicated time points and mice culled at day 28 postdelivery to collect lavage fluid for T1-3B quantification using ELISA. (A) Serum levels of T1-3B presented as group mean and SEM. (B) Levels of T1-3B in the ELF with each data point representing an individual animal and horizontal bars representing group means. The dotted line represents the lowest detection limit of the ELISA. Values plotted as 0.002 µg/mL in B indicates no detectable T1-3B. ELF, epithelial lining fluid; SIV, simian immunodeficiency virus; SEM, SE of the mean; TU, transducing units.

A rSIV.F/HN vector was generated to express T1-3B anti-influenza nAb under the transcriptional control of the hCEF promoter, which is known to be active in the human lung.^6^ For simplicity, the T1-3B IgG1 heavy and light-chain cDNAs were fused into a single open reading frame via a furin cleavage site and 2A self-processing peptide.^2^ Intranasal delivery of this vector (rSIV.F/HN.hCEF.T13B) at doses of 6e5, 6e6 or 5e7 transducing units (TU) resulted in T1-3B in the serum and epithelial lining fluid (ELF, n=6–8). Serum T1-3B was detected as early as 7 days post-delivery, reached steady-state levels after 14 days and persisted, essentially unchanged, to the end of the study (day 28, figure 1A). Previous studies with reporter genes have shown that rSIV.F/HN-mediated lung transgene expression can persist for the lifetime of the animal.^6–8^ Mean treatment group serum T1-3B levels ranged from ~0.1 to ~0.3 µg/mL and were significantly (p<0.001) dependent on vector dose (figure 1A). Higher levels of T1-3B (~2 µg/mL) were observed in the respiratory ELF (p<0.001, figure 1B).

### rSIV.F/HN expression of T1-3B protects mice against supra-lethal influenza challenge

**Figure 2:**
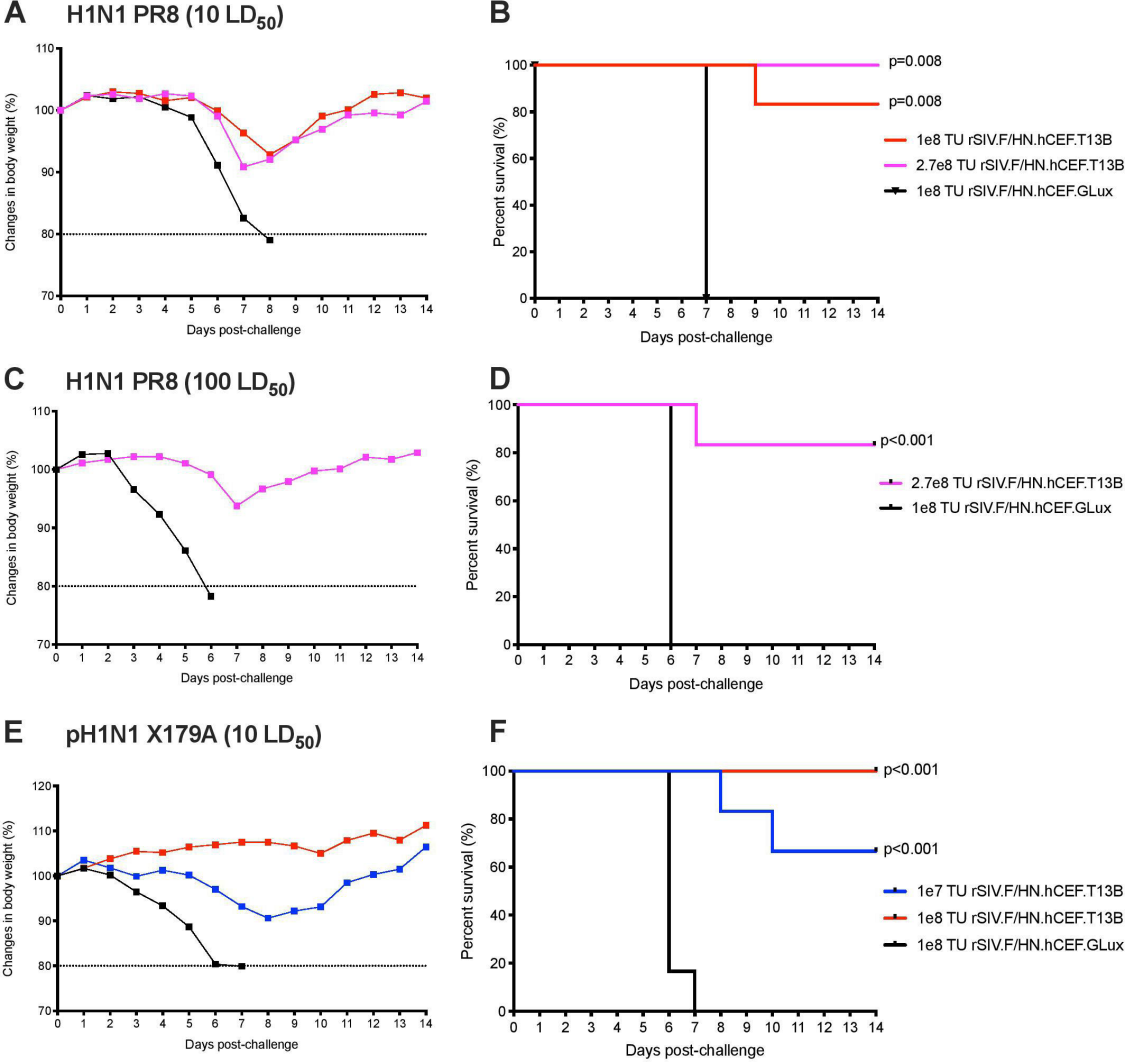
Supralethal influenza challenge in mice expressing T13B antibody via the rSIV.F/HN vector. Mice (n=5–6 per group) were dosed intranasally with rSIV.F/HN.hCEF.T13B at a dose of 1E7 TU (blue), 1e8 TU (red), 2.7e8 TU (magenta) or with 1e8 TU (black) of rSIV.F/HN.hCEF.GLux (*Gaussia Luciferase*) as mock control. (A–D) BALB/c mice were challenged with 10 or 100 median lethal dose (LD_50_) of H1N1 A/PR/8/1934 (Cambridge). (E, F) DBA/2 mice were challenged with 10 LD_50_ of reassortant pandemic H1N1 A/CA/7/2009-X179A. Body weight was measured daily for 14 days and mice were euthanised if weight loss declined ≥20% as depicted in the Kaplan-Meier survival curve (right column). SIV, simian immunodeficiency virus; TU, transducing units.

To assess if rSIV.F/HN vector expressing T1-3B antibody could protect mice against influenza challenge, mice were dosed intranasally (n=5–6) with rSIV.F/HN.hCEF.T13B (1e8 or 2.7e8 TU), or with rSIV.F/HN.hCEF.Glux expressing the *Gaussia luciferase* reporter gene as a negative control (1e8 TU). After 1 month, the mice were challenged with a supralethal dose (10 Median Lethal Dose (LD_50_)) of PR8 strain influenza (H1N1 A/Puerto Rico/8/1934 (Cambridge)). As expected, all mice receiving the negative control vector lost weight rapidly after influenza challenge reaching a humane endpoint (20% wt loss) within 8 days (figure 2A). Mice receiving rSIV.F/HN.hCEF.T13B showed only mild (~10%) weight loss after influenza challenge, resulting in 83% and 100% survival in the 1e8 TU (p=0.08) and 2.7e8 TU (p=0.08) group, respectively (figure 2B). To further investigate the extent of influenza protection, mice (n=6) were dosed with 2.7e8 TU of rSIV.F/HN.hCEF.T13B or 1e8 TU of control rSIV.F/HN.hCEF.Glux vector, and then challenged with a higher dose of PR8 (100 LD_50_). Mice receiving the control vector showed rapid weight loss after influenza challenge reaching the humane endpoint within 6 days, while mice receiving 2.7e8 TU of rSIV.F/HN.hCEF.T13B showed only mild weight loss after influenza challenge, which resulted in 83% survival (p<0.001, figure 2C, D).

To evaluate the potential for rSIV.F/HN expression of T1-3B antibody to provide a broad protection against influenza strains, mice were challenged with 10 LD_50_ of NYMC X-179A, a reassortant H1N1 virus of A/California/7/2009 (the 2009 pandemic influenza, n=6). Mice receiving control vector lost weight rapidly after influenza challenge and reached the humane endpoint within 7 days. Mice in the 1e7 TU rSIV.F/HN.hCEF.T13B dose group experienced only fair weight loss (figure 2E) resulting in 67% survival (p<0.001, figure 2F), but mice receiving a higher dose (1e8 TU of rSIV.F/HN.hCEF.T13B) were completely protected against weight loss and showed 100% survival (p<0.001, figure 2E, F). Together, these findings demonstrate that rSIV.F/HN-mediated expression of T1-3B antibody protects mice against at least two diverse influenza strains.

**Figure 3:**
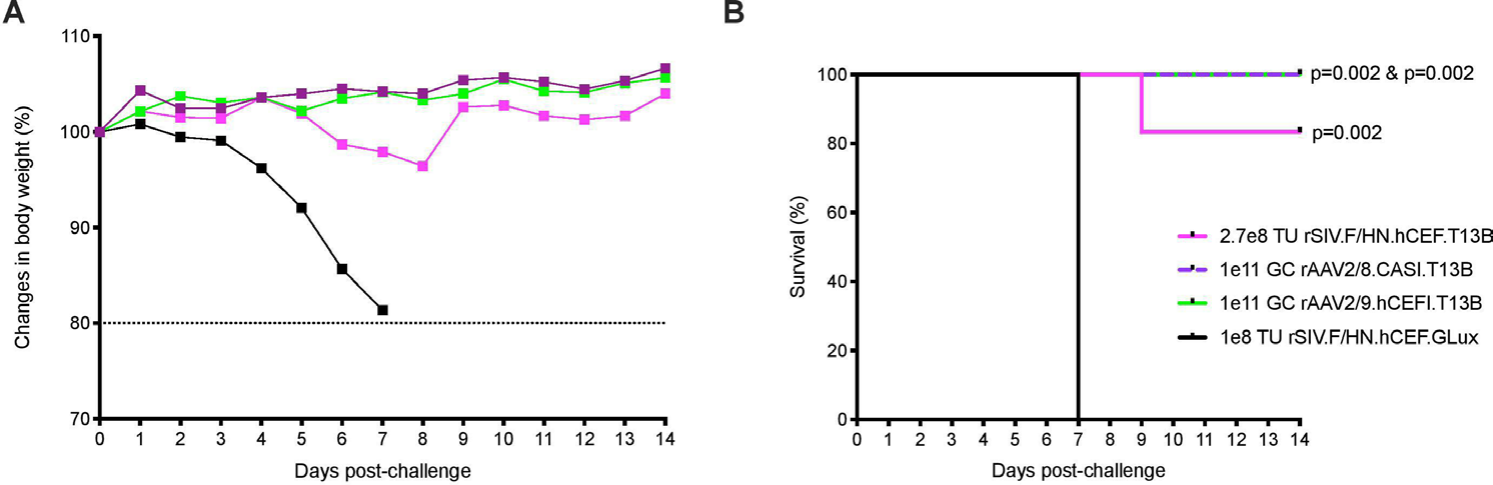
Protection of mice against influenza challenge mediated by rSIV.F/HN is comparable to protection offered by rAAV8 or rAAV9. Mice (BALB/c, n=5–6 per group) were dosed with rSIV.F/HN.hCEF.T13B (2.7e8 TU, magenta), rSIV.F/HN.hCEF.GLux (1e8 TU black) or rAAV2/9.hCEFI.T13B (1e11 GCs, green) via intranasal instillation, or with rAAV2/8.CASI.T13B (1e11 GC, purple) delivered via intramuscular injection. After 1 month, mice were challenged with 10 LD_50_ of H1N1 A/PR/8/1934 (Cambridge). Body weight (A) was measured daily for 14 days and mice were euthanised if weight loss declined ≥20% as depicted in the Kaplan-Meier survival curve (B). GCs, genome copies; rAAV, recombinant adeno-associated virus; SIV, simian immunodeficiency virus; TU, transducing units.

Other viral vectors have also been used to generate influenza passive immunity, so we compared the protection mediated by rSIV.F/HN vector with that provided by rAAV. Groups of mice (n=5–6) were dosed intranasally with rSIV.F/HN.hCEF.T13B (2.7e8 TU), or with rAAV2/9.hCEFI.T13B (1e11 genome copies (GCs)). An additional group received rAAV2/8.CASI.T13B (1e11 GC), comprising an alternative AAV serotype and promoter, via intramuscular delivery. Mice receiving rSIV.F/HN.hCEF.GLux (1e8 TU, intranasal) served as a negative control. All groups of mice were subsequently challenged 1 month postdose with 10 LD_50_ of PR8 influenza. Consistent with the observations of others,^2 3^ mice receiving rAAV vectors either intranasal or intramuscular were completely protected against weight loss (figure 3). Mice receiving the rSIV.F/HN vector experienced very mild weight loss, resulting in 83% survival. The results showed that the rSIV.F/HN vector approach is comparable to the rAAV2/8 and rAAV2/9 vector platforms in providing protection against a supralethal influenza challenge.

## Discussion

The rSIV.F/HN vector is currently in preparation for cystic fibrosis gene therapy clinical trials.^6^ It has also been shown that transduction of rSIV.F/HN can direct expression of secretory proteins, both locally and systemically.^8^ The aim of this study was to investigate delivery of rSIV.F/HN to the lungs to express antibodies for passive immunisation against influenza infection. To our knowledge, this study describes for the first time the use of a lentiviral vector for antibody expression in vivo.

We show that rSIV.F/HN-mediated expression of nAb T1-3B in the murine lung leads to detectable levels of antibody in the lung lumen and serum. The antibody levels are sufficient to protect two mouse strains against lethal challenge with two different influenza strains. We also show that T1-3B antibody expression following intranasal delivery of rSIV.F/HN provides protection against lethal influenza challenge which is comparable to rAAV vectors. Potential advantages offered by the rSIV.F/HN lentiviral vector include a greater capacity for packaging of large transgenes and the ability to repeatedly administer the vector without loss of efficacy. Repeat administration of many viral vectors, particularly rAAV,^9^ has been unsuccessful in the lung due to immune responses against the vector. The demonstration that rSIV.F/HN can be repeat administered to the murine lung without loss of transgene activity,^7^ however, offers the possibility of using this approach to enhance antibody expression, or to tackle new influenza strains as they emerge.

Although larger sample size might be needed to improve statistical power, this study demonstrates the successful use of rSIV.F/HN to mediate passive immunisation against influenza, an approach that could potentially be used in combination with conventional influenza vaccines. It may also have application for timely and longlasting protection of health workers and other essential personnel during emerging pandemics, such as Ebola virus and new strains of COVID-19.
