# Supplementary material for: Lung-targeting lentiviral vector for passive immunisation against influenza
Source: Thorax. 2020 Sep 3;75(12):1112–5. doi: 10.1136/thoraxjnl-2020-214656 (PMC7677466; doi:10.1136/thoraxjnl-2020-214656)
Supplement: Supplementary data [file thoraxjnl-2020-214656supp001.pdf]

Lentiviral vector production

Lentiviral vector (rSIV.F/HN), pseudotyped with the lung-targeting F and HN proteins from Sendai Virus, was produced via five-plasmid transient transfection of HEK293T cells as described [1]. In brief, genome plasmids were constructed expressing a codon-optimised T1-3B bnAb (GenBank: KP231622.1; KP231643.1)[2] in the single-ORF expression configuration described previously [3], or a CpG-free *Gaussia Luciferase* (soGLux), inserted via unique *NheI* and *PsPOMI* sites, and driven by the hCEF promoter [4]. Vectors were purified using anion exchange chromatography and tangential flow filtration and formulated into either FreeStyle293 media or TSSM buffer (tromethamine 20 mM, NaCl 100 mM, sucrose 10 mg/mL, and mannitol 10 mg/mL). The functional titre of lentiviral vectors was determined based on the genomic integration of WPRE DNA sequence. Suspension HEK293F cells were transduced with dilutions of purified lentiviral vectors in FreeStyle293 media in the presence of 8 µg/mL of polybrene. Cells were incubated for 72 hours before genomic DNA was extracted. The integrated WPRE DNA was quantified against a standard curve of DNA mimics containing the WPRE sequence using the following primers: Forward: TGGCGTGGTGTGCACTGT; Reverse: CCCGGAAAGGAGCTGACA; Probe: FAM-TTGCTGACGCAACCCCCACTGG-TAMRA in triplicate using TaqMan FAST Universal Master Mix, normalised against total ng DNA. The PCR profile used was as following: 50ºC for 2 min, 95ºC for 10 min followed by 40 cycles of 95ºC for 15 sec and 60ºC for 1 min. Functional transducing units per mL of virus supernatant (TU/mL) was determined from the slope of a linear regression curve generated from the dilution series.

Recombinant adeno-associated virus vector production

Recombinant rAAV was produced as described [5]. In brief, HEK293T cells were transfected with the plasmids pAdDeltaF6, and pAAV2/8 using Polyethylenimine (PEI; Polysciences Inc.). After 72 hours, cells were resuspended in lysis buffer (1M Tris(hydroxymethyl)aminomethane, 150 mM NaCl) and EDTA-free protease inhibitor cocktail and underwent four freeze-thaw cycles. Cell lysates were incubated (37˚C for 30 min) with Benzonase (50 U/mL final concentration) and clarified via centrifugation, and purified using iodixanol gradient fractionation and diafiltration into D-PBS using Amicon Ultra-15 100K MWCO filters. The number of Genome Copies (GC/ml) was determined by qPCR. Purified rAAV was incubated with DNAse I (100°C for 10 min)

to denature viral capsids and release the viral DNA and qPCR performed using Taqman Fast Universal PCR Master Mix against a standard curve of DNA mimics containing the WPRE sequence using the following primers: Forward: TGGCGTGGTGTGCACTGT; Reverse: CCCGGAAAGGAGCTGACA; Probe: FAM-TTGCTGACGCAACCCCCACTGG-TAMRA. Amplification data generated were used to determine the GC/mL.

Influenza virus production

Influenza (H1N1 A/Puerto Rico/8/1934 (PR8) (Cambridge strain) and X-179A (reassortant virus containing the H1 and N1 from the 2009 pandemic A/California/7/2009 strain and internal genes of PR8) were produced and propagated in MDCK-SIAT1 cells in the presence of TPCK-treated trypsin as described previously [6]. Tissue culture infectious dose ($TCID_{50}$) of the viruses were determined in MDCK-SIAT1 infected with a ½-log dilution series of viruses prepared in VGM (Viral Growth Media; DMEM, 1% BSA, 10 mM HEPES buffer and 100 U/mL penicillin and 100 µg/mL streptomycin) for 1 hour in eight replicates on a 96-well flat-bottomed plate. The cells were then topped with 150 µl of VGM with TPCK-treated trypsin (1 µg/mL) and incubated for 48 hours at 37°C. Infection was determined by staining for anti-NP (virus nucleoprotein) expression in the cells. In house human anti-NP antibody 2-8C was used at 1 µg/ml with the secondary antibody HRP labelled rabbit anti-human IgG (1:1600) (dako P0214). $TCID_{50}$ was calculated using the method of Reed and Muench [7].

In vivo viral vectors transduction and influenza challenge

All procedures involving laboratory mice were carried out at the Biomedical Services Unit (BMS) (University of Oxford, John Radcliffe Hospital, Oxford, UK) in accordance with the UK Home Office approved project and personal licenses under the terms of the Animals (Scientific Procedures) Act 1986 (ASPA 1986). Female BALB/cOlaHsd or DBA/2OlaHsd (for X-179A challenge) mice aged 5 to 7 weeks at point of procedure were purchased from the BMS or Envigo RMS UK. For intranasal dosing, mice were lightly anesthetised by isofluorane and 50 µl or 100 µl of viral vector or influenza virus

was administrated via nasal instillation in a single, continuous droplet with the mouse held vertically with mouth closed. For intramuscular dosing, mice were anaesthetised, the surface of the gastrocnemius muscle was cleaned with 70% ethanol and rAAV vector (in 40 µL) was delivered via injection. Following influenza challenge, mice were monitored daily and mice with weight loss ≥20 percent were euthanized by rising concentration of CO_2. At indicated time-points post dosing, sera and lavage fluid was collected as previously described [8].

Quantification of T1-3B expression

T1-3B is a Group 1 HA (H1, H2 and H5) broad reactive stem-targeting antibody isolated via single antigen-specific plasmablast cloning from vaccinated donor. Expression of T1-3B in mouse sera and lavage fluid was determined using anti-human IgG Enzyme-Linked Immunosorbent Assay (ELISA), performed using Human IgG ELISA kit (Bethyl Laboratories) according to the manufacturer's instructions. In brief, dilutions of serum and neat lavage fluid samples were added to 96-well plates coated with goat anti-human IgG-Fc antibody and blocked overnight. After 1 hour incubation, HRP-conjugated goat anti-human IgG antibody (1:200,000) was added for 1 hour. Plates were read at OD_{450} after developing with 3,3′,5,5′-tetramethylbenzidine (TMB) substrate and reaction stopped with 0.18 M H_2SO_4 solution. The concentration of human IgG in the samples was determined using a standard curve. Expression levels in ELF were corrected for the dilution using lavage fluid collection urea assay as described previously [1].

Statistical analyses.

Differences in serum human IgG concentrations in time-course studies were determined using Dunnett's post-hoc tests after significant ANOVA analyses on area-under-the-curve values calculated for individual animals. Differences in ELF human IgG concentrations at a single time-point were determined using Dunnett's post-hoc tests after significant ANOVA analyses on log-transformed individual animal values. Differences in survival during influenza challenge studies were determined using Kaplan-Meir survival curves and the Mantel-Cox log-rank test. A probability value (p-value) of less than 0.05 was considered statistically significantly different from

unlabelled comparator; exact p-values are presented to three decimal places or indicated as <0.001. Numerical manipulations were performed in Excel (Microsoft, Redmond, USA), statistical analyses were performed using Prism 8.0 (GraphPad, San Diego, USA).

1. Michael C. Paul-Smith, K.M.P., Jean-François Gelinas , Jenny McIntosh, Ian Pringle, Lee Davies, Mario Chan, Cuixiang Meng, Robyn Bell, Lidia Cammack, Caroline Moran ,Loren Cameron, Makoto Inoue, Shu Tsugumine, Takashi Hironaka, Deborah R. Gill, Stephen C. Hyde, Amit Nathwani, Eric W. F. W. Alton, Uta Griesenbach, *The murine lung as a factory to produce secreted intrapulmonary and circulatory proteins.* Gene Therapy, 2018. **23**: p. 345-358.
2. Huang, K.Y., et al., *Focused antibody response to influenza linked to antigenic drift.* J Clin Invest, 2015. **125**(7): p. 2631-45.
3. Balazs, A.B., et al., *Broad protection against influenza infection by vectored immunoprophylaxis in mice.* Nat Biotechnol, 2013. **31**(7): p. 647-52.
4. Hyde, S.C., et al., *CpG-free plasmids confer reduced inflammation and sustained pulmonary gene expression.* Nat Biotechnol, 2008. **26**(5): p. 549-51.
5. Silva, S.R.d., et al., *Adeno-Associated Viral Gene Therapy for Retinal Disorders.* Gene Delivery and Therapy for Neurological Disorders, 2015. **98**: p. 203-228.
6. Powell, T.J., et al., *Pseudotyped influenza A virus as a vaccine for the induction of heterotypic immunity.* J Virol, 2012. **86**(24): p. 13397-406.
7. L.J., R. and M. H., *A simple method of estimating fifty per cent endpoints.* Am. J. Hyg. (Lond.) 1938. **27**: p. 493-497.
8. Griesenbach, U., et al., *Secreted Gaussia luciferase as a sensitive reporter gene for in vivo and ex vivo studies of airway gene transfer.* Biomaterials, 2011. **32**(10): p. 2614-2624.
